# Construction of biological networks from unstructured information based on a semi-automated curation workflow

**DOI:** 10.1093/database/bav057

**Published:** 2015-06-16

**Authors:** Justyna Szostak, Sam Ansari, Sumit Madan, Juliane Fluck, Marja Talikka, Anita Iskandar, Hector De Leon, Martin Hofmann-Apitius, Manuel C. Peitsch, Julia Hoeng

**Affiliations:** ^1^ Philip Morris International R&D, Philip Morris Products S.A., Quai Jeanrenaud 5, 2000 Neuchâtel, Switzerland and; ^2^ Fraunhofer Institute for Algorithms and Scientific Computing, Schloss Birlinghoven, Sankt Augustin, Germany

## Abstract

Capture and representation of scientific knowledge in a structured format are essential to improve the understanding of biological mechanisms involved in complex diseases. Biological knowledge and knowledge about standardized terminologies are difficult to capture from literature in a usable form. A semi-automated knowledge extraction workflow is presented that was developed to allow users to extract causal and correlative relationships from scientific literature and to transcribe them into the computable and human readable Biological Expression Language (BEL). The workflow combines state-of-the-art linguistic tools for recognition of various entities and extraction of knowledge from literature sources. Unlike most other approaches, the workflow outputs the results to a curation interface for manual curation and converts them into BEL documents that can be compiled to form biological networks. We developed a new semi-automated knowledge extraction workflow that was designed to capture and organize scientific knowledge and reduce the required curation skills and effort for this task. The workflow was used to build a network that represents the cellular and molecular mechanisms implicated in atherosclerotic plaque destabilization in an apolipoprotein-E-deficient (ApoE ^−/−^ ) mouse model. The network was generated using knowledge extracted from the primary literature. The resultant atherosclerotic plaque destabilization network contains 304 nodes and 743 edges supported by 33 PubMed referenced articles. A comparison between the semi-automated and conventional curation processes showed similar results, but significantly reduced curation effort for the semi-automated process. Creating structured knowledge from unstructured text is an important step for the mechanistic interpretation and reusability of knowledge. Our new semi-automated knowledge extraction workflow reduced the curation skills and effort required to capture and organize scientific knowledge. The atherosclerotic plaque destabilization network that was generated is a causal network model for vascular disease demonstrating the usefulness of the workflow for knowledge extraction and construction of mechanistically meaningful biological networks.

## Introduction

The volume of scientific knowledge has increased rapidly in the past 50 years. Medline, the most comprehensive bibliographic database in the life sciences, currently indexes more than 5000 journals and contains abstracts of more than 20 million articles ( http://www.nlm.nih.gov/bsd/index_stats_comp.html ). The number of indexed articles per year has grown constantly: e.g. from 9547 in 1960 to 150 031 in 2013 for cancer, and from 257 in 1960 to 7287 in 2013 for atherosclerosis ( http://dan.corlan.net/medline-trend.html ).

Model organism databases (MODs), e.g. WormBase ( 1 ) and the Mouse Genome Informatics (MGI) database ( 2 ), have been developed to store the genetic and genomic data for sequenced species, in this example, mouse and worm. The processes developed for literature searches and biological data extraction leading to systematic information capture and organization, e.g. for gene sequence and function information, are now known as biocuration. Controlled vocabularies or ontologies such as the Gene Ontology (GO) ( http://www.geneontology.org/ ) have been developed to capture the biological data found in literature. These ontologies are used consistently across different MODs and are amenable to computer manipulation. In this context, text mining tools for managing information recognition and extraction have become increasingly relevant ( 3 ). Text mining can help identify genes or proteins and can be used to map them to controlled vocabularies (e.g. gene lists, phenotypes and ontologies). Van Auken *et al* . ( 4 ) showed that Textpresso, a category-based information retrieval and extraction system developed by WormBase, increased curation efficiency by at least 8-fold, and perhaps by as much as 15-fold (given differences in individual curatorial speed) compared with manual curation processes. Using the named entity recognition (NER) tool ProMiner ( 5 ), MGI curators improved their overall efficiency by around 20% without compromising the quality of the curation ( 6 ). Despite the striking progress in biocuration and text mining approaches in the context of curated databases, little progress has been made in writing scientific knowledge in a structured and computable form. Most curated databases are still descriptive in terms of biological entities, where the entities are not interconnected and are not in a computable form. Hence, a new curation challenge is to convert scientific relationships embedded in the biomedical literature into a structured knowledge base ( 7–9 ). Scientific knowledge curated at the system level will help researchers rapidly query, visualize and analyse the specific interaction networks implicated in diseases and open new opportunities for the identification of critical biomedical entities as therapeutic targets ( 10–12 ).

Knowledge curation into computable format requires a well-defined structured and standardized language ( 13 ). The two most popular modeling and data exchange languages currently in systems biology are the biological pathway exchange language (BioPAX) and systems biology markup language (SBML). BioPAX can be used to describe the biological semantics of metabolic, signaling, molecular, gene-regulatory and genetic interaction networks. It has been used mainly for qualitative analysis and information exchange ( 14 ). SBML is a structured eXtensible Markup Language (XML)-based data exchange language that has been used to model biochemical reaction networks, including cell signaling metabolic pathways and gene regulation. Unlike BioPAX, SBML can accommodate mathematical expressions, which are necessary for dynamic simulations ( 15 ).

Recently, Selventa, a biotechnology company with a strong focus on Big Data analyses, released the biological expression language (BEL) ( www.openbel.org ), which allows knowledge modeling in a computable form. BEL can be used to represent biological knowledge captured in causal and correlative relationships that are triplets that contain a subject, a predicate (representing a relationship) and an object. It can be represented in three different machine-readable formats: in XML, resource description framework, and BEL Script. The latter is very similar to human language. BEL was designed to represent discrete scientific findings and their relevant contextual information as qualitative causal relationships that can drive knowledge-based analytics. The language supports the collation and use of scientific findings to assemble models dynamically. The models range from large models that can be considered causal network knowledge base models to small models that represent pathways. A knowledge base encoded in BEL can be used to query, interpret, analyse and visualize networks ( 16–19 ). Relationship information in a computable format serves systems biology approaches, especially the network-based approaches that have emerged as powerful tools for interpreting high-throughput data ( 20 ). For example, a network-based approach was used to explain and predict the development of obesity and type 2 diabetes ( 21 , 22 ). Similarly, Kumar *et al* . ( 23 ) used causal network modeling in a drug development approach to show the anti-proliferative mechanism of action of a novel AKT drug inhibitor.

Here, we present the first application of a novel semi-automated knowledge extraction workflow. The workflow allows the extraction of causal and correlative biological relationships using text mining and automated transcription into a system modeling language ( 24 ). Furthermore, the system provides relevant information to users to help narrow down the captured information into accurate curated statements. As an example of its application, we built a computable knowledge base that represents atherosclerosis plaque destabilization using this workflow. Atherosclerosis plaque destabilization is the main biological process responsible for cardiovascular disease (CVD), the major cause of mortality in industrialized countries. Indeed, the World Health Organization estimated that 17.3 million people died from CVD in 2008, representing 30% of all global deaths ( http://www.who.int/mediacentre/factsheets/fs317/en/ ). Atherosclerosis is a chronic inflammatory disease of large arteries that can lead to fatal complications including myocardial infarction and/or stroke. The mouse apolipoprotein E–deficient (ApoE ^−/−^ ) model of atherosclerosis has been used extensively to investigate human CVD ( 25 ). Early atherogenic mechanisms are amenable to controlled *in vitro* experimental perturbations; however, advanced atherosclerotic lesions and the mechanisms leading to plaque rupture are difficult to recreate experimentally. Disease modeling approaches and information extraction methods constitute invaluable tools to overcome these experimental limitations.

We adopted the semi-automated knowledge extraction workflow and modeled causal and correlative relationships extracted from original scientific publications focused on plaque development and destabilization using the ApoE ^−/−^ model of atherosclerosis. We then compiled the extracted information into a knowledge network model.

## Methods

### The semi-automated workflow

**Figure 1.**
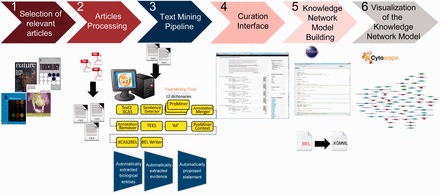
Overall workflow used to create the atherosclerosis plaque destabilization network. The knowledge extraction workflow contains six steps. Step 1: Selection of articles that represent specific biological context from which the knowledge base was constructed. Step 2: Text-processing with special character clearance and line break corrections to ensure proper machine parsing. Step 3: Text mining pipeline with an automated recognition of NER and relationships and coding into a BEL-compliant syntax. Step 4: Domain expert curation process in the curation interface based on the automatically created and proposed BEL statements. Step 5: Validation and transformation of BEL statements into knowledge network models. Step 6: Visualization of the knowledge network model.

The technical details of the knowledge extraction workflow have been described in detail by Fluck *et al* . ( 24 ). A schematic representation of the semi-automated knowledge extraction workflow is shown in Figure 1 . Here, the various steps are described briefly:

#### Step 1: Article selection

Selection of relevant articles that summarize specific biological mechanisms involved in the atherosclerosis plaque destabilization process. We narrowed the scope of the articles to include only scientific findings derived from experiments conducted on mice with the ApoE ^−/−^ genetic background. Keywords, such as ‘atherosclerosis plaque destabilization’, ‘vulnerable lesion’ and ‘advanced lesions’ were used to find relevant scientific articles in the PubMed library ( http://www.ncbi.nlm.nih.gov/pubmed ). We used several review articles that summarized the original findings to guide us to specific articles that accurately described the mechanistic pathways ( 26–31 ).

#### Step 2: Article processing

The pipeline requires a plain text input, which also is a prerequisite for most other text parsing and analysis tools and pipelines. Because most of the selected articles were available only in PDF format and had varying layouts, we converted the PDF files into text files with the ABBYY FineReader 11 ( http://www.abbyy.com/finereader/ ). To retrieve a large number of causal relationships from these documents we extracted text from the abstract, material and method and result sections ( 32 , 33 ). Relevant passages were selected manually to avoid possible conversion errors and manual corrections. We excluded the introduction, discussion and conclusion sections because these sections typically contain non-causal evidence, repetition of the results and descriptions of non-factual hypotheses. Tables and graphics were excluded because of the technical challenges involved in correctly parsing these objects.

#### Step 3: Text mining pipeline

Recognizing relevant biological terms in a text is fundamental for semantic retrieval and extraction of relationships. The pipeline, which was based on the Unstructured Information Management Architecture (UIMA) ( https://uima.apache.org/ ), integrated and combined linguistic algorithms for NER and relationship extraction. A text document collection given as input to the pipeline can be output as XML-based BEL (XBEL) documents, an XML version of BEL.

For dictionary-based NER, the pipeline features ProMiner, which was shown to be efficient in BioCreative NER assessments ( 5 , 34 ), and integrates several dictionaries that have been optimized for use in systems biology ( 24 ). The evaluation of ProMiner NER for human and mouse gene/protein names achieved F-scores of 0.79 (for human) and 0.8 (for mouse). The NER performances of a number of dictionaries tested in the current workflow for relationship extraction of protein function inhibitors are shown in Table 3 .

The Turku Event Extraction System (TEES), a support vector machine-based text mining system developed at the University of Turku, Finland ( 35 ), was integrated for the extraction of events and relations from natural language text sources. The NER performance of TEES was tested within the biomedical text mining (BioNLP shared task) ( http://2011.bionlp-st.org/ ) assessments and reached an overall F-score of 53.3%. Relationships detected by TEES are output as ‘positive regulation’ and ‘negative regulation’ and are mapped and translated to BEL via a BEL converter within the UIMA pipeline. Further details about the conversion process have been described by Fluck *et al.* ( 10 ). The BEL converter creates BEL statements and generates two documents (XBEL and XMI) as output. The XBEL facilitates its transformation into an assembled knowledge network model. It contains evidence, statements and annotations (e.g. name spaces, experimental context and publication details) to fully describe the BEL statements ( Supplementary File S1 ). The XMI document contains information that cannot be coded in BEL but is relevant for the curation interface (e.g. text location of the recognized entities and alternative namespaces).

#### Step 4: Curation interface

**Figure 2.**
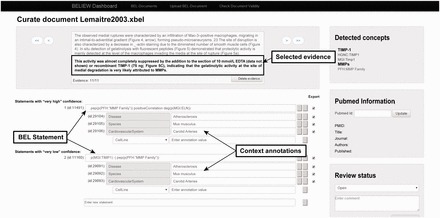
Screenshot of the knowledge extraction curation interface.

The workflow contains an interface that allows manual modification of the automatically generated XBEL documents by employing the additional information from the XMI document. The interface gives the user access to the evidence text and highlights the results, such as named entities and their namespaces, obtained from the previous text mining pipeline ( Figure 2 ). Each processed text document can be opened in the curation interface, and users can read each piece of selected evidence and review the specific scientific relationships that have been represented as BEL-compliant statements. After modification and curation each BEL statement is re-validated automatically to guarantee compliant BEL syntax.

The curation interface was developed specifically to cover the needs of users with little curation experience. Therefore an evidence-centric display of the statements is generated during the curation process; that is, for each piece of evidence within the document, all full or fragmented causal relationships recognized by the text mining pipeline are listed as BEL statements. Fragmented BEL statements (e.g. subject-relationship or relationship-object) can be assembled by the user to form new complete BEL statements. Each BEL statement is specified by context information: in this case, tissue, disease, cell and species. The interface preserves the original XBEL document and creates a copy of the modified XBEL document based on the curation activity. After it is saved, a syntax validation is performed based on a validator in the BEL framework toolset and integrated into the curation interface.

#### Step 5: Building the knowledge network model

The verified BEL documents were used in the BEL framework toolset, which includes a compiler to generate a knowledge network model, which is in this case, a knowledge assembly model (KAM), for each BEL document. Several BEL documents were then compiled into a single KAM and either hosted or exported into the eXtensible Graph Markup and Modeling Language (XGMML) format, Cytoscapes’ standard format for saving graph layouts. The KAMs were used as the knowledge base of structured scientific information for atherosclerosis plaque destabilization.

#### Step 6: Visualization of the knowledge network model

The KAM navigator, another application in the BEL framework, is a KAM-hosting daemon that can be accessed via Cytoscape ( 36 ) and allows the KAM to be exported into XGMML format for visualization and network analysis. Cytoscape is used to visualize the KAM in a dynamic view. The system also allows to integrate the network with any type of attribute data and use various tools for network analysis.

### Evaluation of the semi-automated knowledge extraction workflow

To assess the efficiency of the semi-automated knowledge extraction workflow compared with conventional manual extraction, two independent users annotated seven full-text articles ( 37–43 ). Both annotators were scientists familiar with BEL. The target was to extract causal and correlative relationships that were specified and demonstrated experimentally in the articles and code them into BEL. No further annotation guidelines or training were provided. One annotator extracted the relationships from the text manually without further support, while the second annotator extracted the knowledge via the semi-automated knowledge extraction workflow. The curation time, the number of BEL statements and the context annotations were recorded for each article and setting (manual vs. semi-automated). In a second step, we evaluated and compared the resulting networks, the curation time and the overlap of manually and automatically extracted evidence.

## Results

The efficiency of the semi-automated curation workflow and manual knowledge extraction was evaluated ( Table 1 ) and the results showed that the semi-automated knowledge extraction workflow took less time than the conventional manual extraction (395 min (∼6 h) for semi-automated vs. 613 min (∼10 h) for manual). We also found that the numbers of BEL statements and annotations retrieved using the semi-automated curation pipeline were higher than those retrieved by manual extraction (234 statements and 112 annotations for semi-automated against 191 statements and 46 annotations for manual) ( Table 1 ). Furthermore, the overall curation time as well as the curation time per BEL statement was almost halved (1.7 min per statement for semi-automated and 3.2 min per statement for manual) or even lower when the annotations also are considered (1.2 min per statement for semi-automated knowledge extraction with annotations and 2.6 min for the manual knowledge extraction with annotations). When the extracted evidence was compared, we found that only 7% of the sentences among the manual and semi-automatically extracted sentences overlapped. Even when the statements were extracted from the same sentences, partly different statements were produced. An example of such differently coded BEL statements is given in the following example where the evidence was extracted from PMID: 21120482 ( 43 ): ‘capillary vessel counting in and around primary tumors showed that CYP4A11 transfection significantly increased microvessel density per high-powered fields (HPF) (34.1 ± 7.3/HPF in control and 35.32 ± 6.4/HPF in GFP group vs. 63.8 ± 11.4/HPF in A549-CYP4A11 group, *P* < 0.05)’.

**Table:** 

Semi-automated:	*p(HGNC:CYP4A11) -> bp(GOBP:angiogenesis)*
Manual:	*p(HGNC:CYP4A11) -> (sec(a(CHEBI:‘20-HETE’)) -> bp(GOBP: ‘blood vessel development’))*

**Table 1.** Content of the two networks created with the semi-automated and manual knowledge extraction processes.

Biological entities	Semi-automated knowledge extraction	Manual knowledge extraction
Number of statements	234	191
Number of annotations	112	46
Overall curation time [min]	395	613
Curation time per statement [min]	1.7	3.2
Curation time per statement + annotation [min]	1.2	2.6
Number of nodes	149	145
Number of edges	285	251

Nodes	Degree of distribution of the most connected nodes	
*p(HGNC:ABL1)*	13	13
*p(MGI:Col4a3)*	13	13
*p(HGNC:FGF2)*	14	14
*Sec(a(CHEBI:‘20-HETE’))*	—	13
*P(HGNC:CYP4A11)*	14	14
*p(HGNC:HMGB1)*	16	16
*p(HGNC:VEGFA)*	19	19
*p(HGNC:THBS1)*	16	16
*A(SCHEM: tumstatin)*	13	—

The semi-automated and manually created networks contain 149 and 145 nodes that are connected by 285 and 251 edges, respectively. Overall curation time, time per statement, and the time per statement and annotation was calculated. The topological analysis of the degree of distribution for both networks showed that the most highly connected nodes (from 13° to 19° of distribution) were similar—namely, VEGFA, HMGB1, THBS1, FGF2, CYp4A11, Col4a3 and ABL1. Only two of the connected nodes, 20-HETE and Tumstatin, were different in the two networks.

Both statements in this example are biologically correct but use different biological processes. In the semi-automated annotation, the statement is aligned to the information in the original sentence, while in the manual annotation, more precise knowledge gathered from the full document is used (secretion of *CHEBI:* ‘ *20-HETE* ’ in the given example). Thus, the level of precision and use of the different biological processes are general discrepancies between the two annotations. To obtain an automated comparison of the networks, we performed a topological analysis of the semi-automated and manual knowledge extracted networks using the Network Analyzer Plugin in Cytoscape ( http://apps.cytoscape.org/apps/networkanalyzer ). The results confirmed the ‘scale-free properties’ of the two networks with comparable properties. Scale-free properties of a network were first described by Barabasi and Bonabeau ( 44 ) and Albert ( 45 ) and are defined by an abundant presence of poorly connected nodes and a low frequency of highly connected nodes, which is a central characteristic of most biological networks ( 45 ). Connections between nodes can be defined by the degree of distribution that indicates the number of times a node is connected to other nodes. Nodes with degrees of distribution that are higher than the average are called hubs ( 46 ). It has been proposed that hub nodes play important roles in a network ( 47 , 48 ). As shown in Table 1 , seven of the most connected nodes are the same for both networks and show comparable connectivity. The *sec(a(CHEBI:* ‘ *20-HETE* ’ *))* node has a larger number of connections (13 connections for *CHEBI:* ‘ *20-HETE* ’ in the manual and seven connections in the semi-automated). In the network generated semi-automatically an additional hub node *a(SCHEM: tumstatin)* was detected. In general, these results show that the semi-automated process with reduced curation time generated networks that were similar to those generated by manual curation. The discrepancies in the statements and the resulting networks are a result of the different BEL coding approaches used in the semi-automated and manual extractions. Clear annotation guidelines may have prevented these deviations and led to a closer match between the two networks produced by the two approaches.

### Application of BEL network generation

To demonstrate the benefits of the semi-automated knowledge extraction workflow a case study based on atherosclerosis plaque destabilization was defined and a knowledge network model was constructed ( Supplementary Figure S1 ). Here, the knowledge network model was assessed and the biological relevance of the network that was generated using our workflow was examined.

The scientific data based on the ApoE ^−/−^ mouse model, a well-established model for human atherosclerosis ( 49 , 50 ), include findings in mice with or without specific regimes or diets. To build a network model that captured the biological mechanisms controlling plaque destabilization, all related causal and correlative relationships (with no boundaries based on tissue context) described in the selected articles were captured. In most studies, the causal and correlative relationships were from the cardiovascular system (aorta, aortic arch and carotid arteries) or from body fluids (blood, plasma and serum).

#### Content of the atherosclerosis plaque destabilization network

The atherosclerotic plaque destabilization network ( Supplementary Figure S1 ) created from the BEL statements that were extracted from filtered articles contains 304 nodes and 743 edges supported by 33 PubMed referenced articles (curated articles are listed in the Supplementary References ). The network consists of 114 protein abundances and 42 RNA abundances as well as 43 biological processes involved in atherosclerosis plaque destabilization in ApoE ^−/−^ mice ( Table 2 ). Relationships between biological entities in the network were represented by edges that were classified broadly as causal and non-causal edges. Causal edges represent directional cause-and-effect relationships between biological entities and are characterized by relationships such as ‘decrease’, ‘increase’, ‘directlyDecrease’ and ‘causesNoChange’. Non-causal edges connect different forms of biological entities, such as protein and RNA without any causal relationship. The workflow identified 566 of the edges as causal and 177 as non-causal relationships. Of the 566 causal edges, 101 showed downregulation (i.e. ‘decrease’) and 299 showed upregulation (i.e. ‘increase’) of the target entity ( Table 2 ).

**Table 2.** Summary of the contents of the atherosclerosis plaque destabilization network.

BEL function	Number of nodes
Abundance (chemicals or lipids)	33
Protein abundance	114
RNA abundance	42
Gene abundance	1
Complex abundance	17
Composite abundance	4
Molecular activity	2
Peptidase activity	8
Kinase activity	6
Catalytic activity	4
Transcriptional activity	1
Degradation	4
Cell secretion	12
Biological process	43
Pathology	13

BEL relationship	Number of edges
Decrease	97
Increase	299
CausesNoChange	166
DirectlyDecrease	4
Causal relationships	566
Non-causal relationships	177

**Table 3.** Evaluation of the efficiency of BEL text mining.

Dictionary	Overall entity count in gold standard	Recall rate initial version (%)	Recall rate final version (%)
Genes/protein (HGNC)	1673	80	93
Chemical compounds (ChEBI)	218	15	66
Chemical compounds (SCHEM)	575	30	75
Chemical compounds (ChEBI + SCHEM + ChEMBL)	793	Not determined	91
Selventa-human-complex	45	40	46
GO-complex	45	Not determined	64
Selventa-human-complex + Complex	45	Not determined	82
GO-function	66	22	Not determined
Selventa-human-families	66	8	77

The recall rate statistics (i.e. the numbers of existing BEL names that were detected) are shown as well as the counts in the manually annotated reference corpus composed of 1348 sentences and 2577 annotations.

#### Topological analysis based on the degree of distribution of nodes

**Figure 3.**
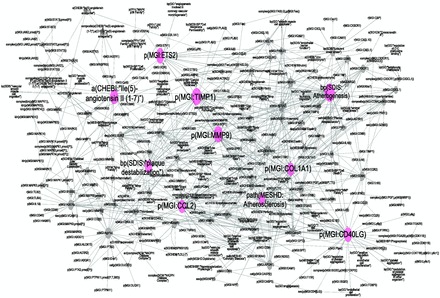
Atherosclerosis plaque destabilization network showing the degree of distribution of nodes. Biological Entities or Nodes. Pink circles indicate the 10 most connected nodes (from 41° to 28°) defined as hubs. From left to right, the hub nodes are Ile2-angiotensin II ( 1-7 ), plaque destabilization, ETS2, CCL2, TIMP1, MMP9, atherosclerosis, COL1A1, atherogenesis and CD40LG. Relationships or edges. Gray lines with arrows indicate positive causal relationships; fine dotted lines with Ts indicate negative causal relationships; gray sine waves indicate correlative relationships; and fine dotted lines indicate non-causal relationship.

Topological analysis of the atherosclerotic plaque destabilization network employing the Network Analyzer Plugin in Cytoscape, confirmed the scale-free properties of the network. When the connections between the nodes in our network were analysed, we observed many poorly connected nodes (average degree of distribution was about 4°) and a few highly connected nodes, the hubs. Biological processes such as ‘atherogenesis’, ‘plaque destabilization’ and ‘atherosclerosis’ were among the 10 most highly connected nodes ( Figure 3 ). Six of the 10 most connected hub nodes (from 41° to 28° of distribution) were the CD40 ligand (CD40LG), the chemokine ligand CCL2, collagen COL1A1, tissue inhibitor of metalloproteinase 1 (TIMP1), avian leukemia oncogene 2 (ETS2) and the matrix metallopeptidase 9 (MMP9) ( Figure 3 ); one of the hub nodes was Ile2-angiotensin II ( 1-7 ), a potent inhibitor of the AT-1 receptor. The high degrees of distribution for these protein nodes suggested that they may have important regulatory roles in the atherosclerosis plaque destabilization network. The CD40LG node had the largest degree of distribution ( 41 ), suggesting a central role for this ligand in the atherosclerosis plaque destabilization network. The CD40LG gene is a member of the tumor necrosis factor gene superfamily that is expressed mainly on CD4+ T cells, platelets, monocytes, macrophages, B cells and endothelial cells. When CD40LG binds to its receptor, it was found to promote the recruitment of leukocytes to lesions, favor inflammation and potentially promote atherogenesis and plaque destabilization ( 47 , 51 , 52 ).

#### CD40LG interacts with other hub nodes

**Figure 4.**
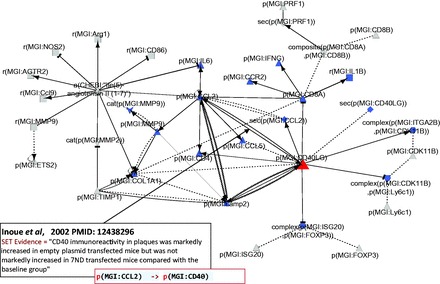
Part of the network showing CD40LG and its interactions with other hub nodes. Biological entities or nodes. CD40LG is indicated in red, and the nodes that are regulated by CD40LG are indicated in blue. An example of evidence extracted from Inoue *et al* . ( 53 ) (PMID: 12438296) with the semi-automated extraction workflow and the associated BEL statement are given in the two boxes on the bottom left of the figure. Square, RNA abundance; triangle, protein abundance; V shape, protein activity; hexagon, complex; diamond, secretion. (B) Relationships or edges. Lines with dark arrows indicate positive causal relationships; lines with dark Ts indicate negative causal relationships; black sine waves indicate correlative relationships and black dotted lines indicate non-causal relationships.

The network shows upregulation of ‘cell adhesion’, ‘hemorrhage’, ‘atherogenesis’ and ‘platelet aggregation’ by CD40LG, which is consistent with its reported proatherogenic and inflammatory effect ( 53 ). Additionally, the CD40LG interactions show a general trend towards pro-inflammatory signaling with an increase in the inflammatory chemokines CCL2 and CCL5 associated with an increase of macrophages and TH1 T cell markers. Indeed, our atherosclerotic plaque destabilization network highlights the upregulation of the CD4 T cell glycoprotein by CD40LG via both a direct action and an indirect CCL2 pathway as well as the simultaneous downregulation of the CD8 T cell glycoprotein by CD40LG ( Figure 4 ). These results suggest that the network recapitulates the important role of adaptive immunity and CD40LG in atherosclerosis plaque progression and destabilization reported previously ( 54 , 55 ). Apart from the inflammatory pathways, the other highly connected hub nodes, CO1A1, TIMP1, ETS2 and MMP9, were found to be associated with extracellular matrix reorganization or degradation in the atherosclerotic plaque destabilization network.

## Discussion and Outlook

In this work we describe a workflow that consists of an automated BEL text mining pipeline and a curation interface that can simplify and accelerate knowledge extraction and representation from published literature. The novelty of this work resides in the workflow, which is a valuable tool for users with little curation experience rather than for experienced curators. This was achieved mainly by using BEL and a curation interface that consolidates relevant information and allows the curation to be performed at the assembled knowledge level instead of the more commonly used entity recognition level.

The relevance of this work is supported by the growing number of publications and unstructured knowledge sources that are now available, making it imperative to develop ways to code biological knowledge in a computable format. The linguistic tools (e.g. ProMiner and TEES) that have been implemented in our text mining pipeline support this process and combine a high recall of biomedical entity relationships with a manual curation process to ensure high precision in knowledge extraction. The semi-automated text mining pipeline extracts and converts causal and correlative relationships between biomedical entities into BEL statements that can be reviewed and corrected by the user. Combined with the human-readable knowledge coding language, BEL allows curation to be performed by any user with knowledge in the biological domain. The underlying technology and curation process scales well with increasing numbers of relevant articles.

As proof of concept, we demonstrated the efficiency of the process by constructing knowledge network models that were visualized using freely available software. The atherosclerosis plaque destabilization network model that was generated using this workflow consists of 304 nodes and 743 edges representing scientific details in a structured cause-and-effect BEL format that also can be read by non-experts because of its strong similarity to natural scientific language. The atherosclerosis plaque destabilization network accurately described the important role of CD40LG in the regulation of chemotaxis, the immune and inflammatory responses, and extracellular matrix disassembly ( Supplementary Figure S1 ).

The semi-automated knowledge extraction workflow presented here detected biological entities (e.g. genes and proteins) with high recall; however, the detection of lipids and chemical compounds was less efficient. The recall rate for gene and protein names was ∼93%, but only 66% and 75% for chemical compounds in the Chemical Entities of Biological Interest (ChEBI) dictionary and SCHEM (OpenBEL names for chemicals, drugs, and other molecule abundances that have not yet been mapped to any other namespace), respectively ( Table 3 ).

To assess the efficiency of the semi-automated knowledge extraction workflow, we compared the manual and semi-automated curation processes using seven randomly selected full text publications ( 37–43 ). A comparison of the resulting networks showed that the two extraction processes produced overall similar networks that contained the same hub proteins, although compared with the manual process, the semi-automated process generated a higher number of nodes, edges and annotations ( Table 1 ). The assisted curation time was approximately half (40–54%) that of the manual curation time. However, the methodology underlines the relevance of annotation guidelines because knowledge statements are expected to deviate from curator to curator, even when the same methodology is used.

Current efforts are focused on assisting users to format tables and lists into BEL statements because these knowledge sources are structured differently from natural language text. Extending the system by incorporating this technology, we expect to see a significant increase in recall. To further improve the rate of recall and automated entity recognition, additional dictionaries with more exhaustive lists of compounds (e.g. lipids) together with suitable normalization methods need to be implemented into the text mining pipeline. Moreover, custom dictionaries, based on namespace gaps identified during the curation process, should also be developed to improve biomedical entity recognition and knowledge extraction.

In the next generation of the text mining pipeline, the system will be enhanced by implementing the best-performing TEES 2.1 ( 56 ), which originally was designed and evaluated in the BioNLP Shared Task 2013. TEES 2.1 now includes models for the recognition of relationships in pathways (especially protein-chemical compound relationships) and models trained specifically for relations between proteins or chemicals and biological processes. These improvements will likely result in a significant increase in the precision of relationship identification.

Computable BEL, which was the main facilitator for the semi-automated creation of knowledge network models, has also been used to interpret ‘omics’ data ( 18 , 20 , 57 ). It is clear that the creation of computable networks representing the current understanding of biological processes is a crucial step in analyzing systems biology and systems toxicology data, which are characterized by multi-dimensional high-throughput data (Big Data). Various algorithms have been developed for the computational analysis of Big Data in biological networks and to quantify network perturbations. The network presented here can be populated with a downstream layer that allows reverse causal reasoning ( 20 ) or network perturbation amplitude can be calculated ( 18 , 58 ) on high-throughput gene expression data. The text mining pipeline can be tested through a web service via the URL: http://www.scaiview.com/belief

## Conclusions

Creating structured knowledge from unstructured text in publications is an important step for the reusability of knowledge. Here, we described a semi-automated knowledge extraction workflow that was designed to reduce curation effort and increase recall of causal relationships compared with manual curation. By curating directly at the knowledge level using a knowledge syntax that is close to human language, the curation task was shifted from the expert curator to users with biological domain expertise. The system is scalable, which will allow it to keep pace with the rapidly increasing number of publications. Moreover, the structured knowledge is computable, which will help in the interpretation of Big Data. The plaque destabilization network presented here provides an easily accessible representation of the main vascular events that lead to plaque rupture and its complications.

## Funding

This work was funded by Philip Morris International.

*Conflict of interest* . None declared.

## Supplementary Material

Supplementary Data
