# Supplementary material for: Construction of biological networks from unstructured information based on a semi-automated curation workflow
Source: Database (Oxford). 2015 Jun 16;2015:bav057. doi: 10.1093/database/bav057 (PMC5630939; doi:10.1093/database/bav057)
Supplement: Supplementary Data [file bav057_Supplementary_Data.zip › Supplementary references list for curated articles.docx]

Supplementary references list for curated articles. Thirty three references papers from which the extracted knowledge were obtained.

1. Aono, J., et al., *Deletion of the angiotensin II type 1a receptor prevents atherosclerotic plaque rupture in apolipoprotein E-/- mice.* Arterioscler Thromb Vasc Biol, 2012. **32**(6): p. 1453-9.

2. Cheng, C., et al., *Heme oxygenase 1 determines atherosclerotic lesion progression into a vulnerable plaque.* Circulation, 2009. **119**(23): p. 3017-27.

3. Cheng, C., et al., *Ets2 determines the inflammatory state of endothelial cells in advanced atherosclerotic lesions.* Circ Res, 2011. **109**(4): p. 382-95.

4. Cheng, C., et al., *Shear stress-induced changes in atherosclerotic plaque composition are modulated by chemokines.* J Clin Invest, 2007. **117**(3): p. 616-26.

5. Cheng, C., et al., *Atherosclerotic lesion size and vulnerability are determined by patterns of fluid shear stress.* Circulation, 2006. **113**(23): p. 2744-53.

6. Clarke, M.C., et al., *Apoptosis of vascular smooth muscle cells induces features of plaque vulnerability in atherosclerosis.* Nat Med, 2006. **12**(9): p. 1075-80.

7. Combadiere, C., et al., *Decreased atherosclerotic lesion formation in CX3CR1/apolipoprotein E double knockout mice.* Circulation, 2003. **107**(7): p. 1009-16.

8. de Nooijer, R., et al., *Lesional overexpression of matrix metalloproteinase-9 promotes intraplaque hemorrhage in advanced lesions but not at earlier stages of atherogenesis.* Arterioscler Thromb Vasc Biol, 2006. **26**(2): p. 340-6.

9. de Nooijer, R., et al., *Overexpression of IL-18 decreases intimal collagen content and promotes a vulnerable plaque phenotype in apolipoprotein-E-deficient mice.* Arterioscler Thromb Vasc Biol, 2004. **24**(12): p. 2313-9.

10. de Vries, M.R., et al., *Plaque rupture complications in murine atherosclerotic vein grafts can be prevented by TIMP-1 overexpression.* PLoS One, 2012. **7**(10): p. e47134.

11. Fukumoto, Y., et al., *Genetically determined resistance to collagenase action augments interstitial collagen accumulation in atherosclerotic plaques.* Circulation, 2004. **110**(14): p. 1953-9.

12. Harja, E., et al., *Vascular and inflammatory stresses mediate atherosclerosis via RAGE and its ligands in apoE-/- mice.* J Clin Invest, 2008. **118**(1): p. 183-94.

13. Inoue, S., et al., *Anti-monocyte chemoattractant protein-1 gene therapy limits progression and destabilization of established atherosclerosis in apolipoprotein E-knockout mice.* Circulation, 2002. **106**(21): p. 2700-6.

14. Johnson, J.L., et al., *Divergent effects of matrix metalloproteinases 3, 7, 9, and 12 on atherosclerotic plaque stability in mouse brachiocephalic arteries.* Proc Natl Acad Sci U S A, 2005. **102**(43): p. 15575-80.

15. Kyaw, T., et al., *Cytotoxic and proinflammatory CD8+ T lymphocytes promote development of vulnerable atherosclerotic plaques in apoE-deficient mice.* Circulation, 2013. **127**(9): p. 1028-39.

16. Lemaitre, V., P.D. Soloway, and J. D'Armiento, *Increased medial degradation with pseudo-aneurysm formation in apolipoprotein E-knockout mice deficient in tissue inhibitor of metalloproteinases-1.* Circulation, 2003. **107**(2): p. 333-8.

17. Leroyer, A.S., et al., *CD40 ligand+ microparticles from human atherosclerotic plaques stimulate endothelial proliferation and angiogenesis a potential mechanism for intraplaque neovascularization.* J Am Coll Cardiol, 2008. **52**(16): p. 1302-11.

18. Li, R., et al., *A dynamic model of calcific nodule destabilization in response to monocyte- and oxidized lipid-induced matrix metalloproteinases.* Am J Physiol Cell Physiol, 2012. **302**(4): p. C658-65.

19. Lievens, D., et al., *Platelet CD40L mediates thrombotic and inflammatory processes in atherosclerosis.* Blood, 2010. **116**(20): p. 4317-27.

20. Lucerna, M., et al., *Vascular endothelial growth factor-A induces plaque expansion in ApoE knock-out mice by promoting de novo leukocyte recruitment.* Blood, 2007. **109**(1): p. 122-9.

21. Luttun, A., et al., *Loss of matrix metalloproteinase-9 or matrix metalloproteinase-12 protects apolipoprotein E-deficient mice against atherosclerotic media destruction but differentially affects plaque growth.* Circulation, 2004. **109**(11): p. 1408-14.

22. Najafi, A.H., et al., *A new murine model of stress-induced complex atherosclerotic lesions.* Dis Model Mech, 2013. **6**(2): p. 323-31.

23. Ni, M., et al., *Atherosclerotic plaque disruption induced by stress and lipopolysaccharide in apolipoprotein E knockout mice.* Am J Physiol Heart Circ Physiol, 2009. **296**(5): p. H1598-606.

24. Olivon, V.C., et al., *Arginase inhibition prevents the low shear stress-induced development of vulnerable atherosclerotic plaques in ApoE-/- mice.* Atherosclerosis, 2013. **227**(2): p. 236-43.

25. Reimers, G.J., et al., *Inhibition of rupture of established atherosclerotic plaques by treatment with apolipoprotein A-I.* Cardiovasc Res, 2011. **91**(1): p. 37-44.

26. Rodgers, K.J., et al., *Destabilizing role of cathepsin S in murine atherosclerotic plaques.* Arterioscler Thromb Vasc Biol, 2006. **26**(4): p. 851-6.

27. Roncal, C., et al., *Short-term delivery of anti-PlGF antibody delays progression of atherosclerotic plaques to vulnerable lesions.* Cardiovasc Res, 2010. **86**(1): p. 29-36.

28. Shipley, J.M., et al., *Metalloelastase is required for macrophage-mediated proteolysis and matrix invasion in mice.* Proc Natl Acad Sci U S A, 1996. **93**(9): p. 3942-6.

29. Silence, J., D. Collen, and H.R. Lijnen, *Reduced atherosclerotic plaque but enhanced aneurysm formation in mice with inactivation of the tissue inhibitor of metalloproteinase-1 (TIMP-1) gene.* Circ Res, 2002. **90**(8): p. 897-903.

30. von der Thusen, J.H., et al., *Induction of atherosclerotic plaque rupture in apolipoprotein E-/- mice after adenovirus-mediated transfer of p53.* Circulation, 2002. **105**(17): p. 2064-70.

31. Yang, J.M., et al., *Angiotensin-(1-7) dose-dependently inhibits atherosclerotic lesion formation and enhances plaque stability by targeting vascular cells.* Arterioscler Thromb Vasc Biol, 2013. **33**(8): p. 1978-85.

32. Yi, G.W., et al., *Overexpression of CXCL16 promotes a vulnerable plaque phenotype in Apolipoprotein E-Knockout Mice.* Cytokine, 2011. **53**(3): p. 320-6.

33. Zadelaar, A.S., et al., *Increased vulnerability of pre-existing atherosclerosis in ApoE-deficient mice following adenovirus-mediated Fas ligand gene transfer.* Atherosclerosis, 2005. **183**(2): p. 244-50.
