# Supplementary figures and images for: Construction of biological networks from unstructured information based on a semi-automated curation workflow
Source: Database (Oxford). 2015 Jun 16;2015:bav057. doi: 10.1093/database/bav057 (PMC5630939; doi:10.1093/database/bav057)

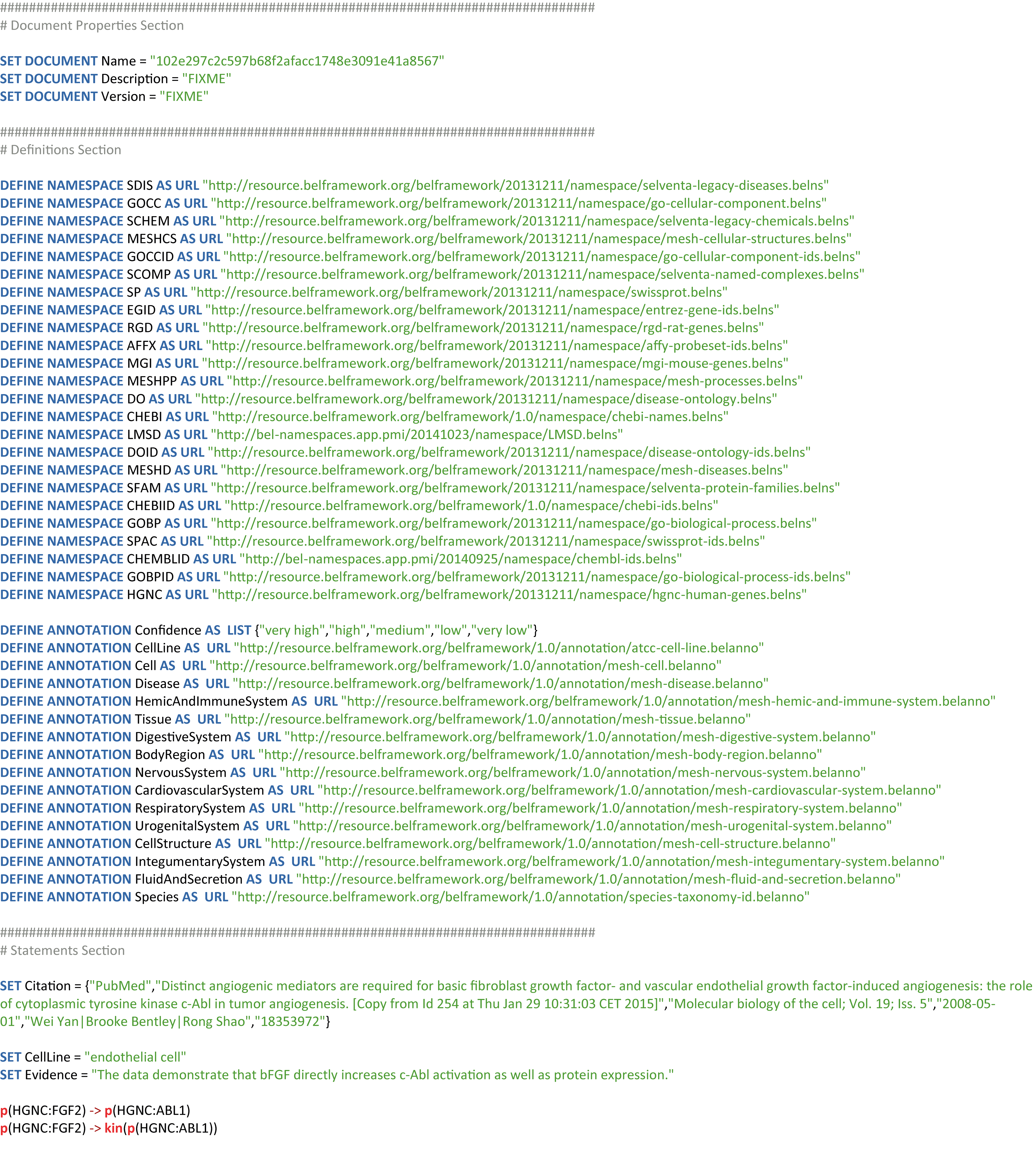

Supplement: Supplementary Data [file bav057_Supplementary_Data.zip › Supplementary File 1(WN).png]
